# *Listeria monocytogenes* Peritonitis in a Patient Receiving Continuous Ambulatory Peritoneal Dialysis: A Case Report and Review of the Literature

**DOI:** 10.1155/2021/6681629

**Published:** 2021-01-28

**Authors:** Ho-Kwan Sin, Au Cheuk, William Lee, Ka-Fai Yim, Clara Poon, Ivy Wong, Bosco Lam, Hon-Lok Tang, Samuel K. Fung

**Affiliations:** ^1^Kwong Wah Hospital, Hong Kong; ^2^Princess Margaret Hospital, Hong Kong

## Abstract

*Listeria monocytogenes* is a rare cause of peritoneal dialysis-related peritonitis. Only a handful of cases have been reported, and the optimal management is still uncertain. We present a case of *Listeria monocytogenes* peritonitis and perform a review of the literature to elucidate optimal antibiotic therapy.

## 1. Introduction

*Listeria monocytogenes* is a Gram-positive bacillus that causes self-limiting disease in healthy subjects [1]. Risk factors for invasive infection include immunocompromised states, old age, malignancy, liver cirrhosis, pregnancy, diabetes mellitus, and alcoholism [2]. Rarely, *Listeria monocytogenes* can cause peritoneal dialysis- (PD-) related peritonitis. Optimal management of *Listeria* peritonitis in PD patients is uncertain.

## 2. Case Report

We present a case of a 49-year-old male patient with end-stage renal disease due to lupus nephritis who had undergone continuous ambulatory PD for 1 year, without previous episodes of peritonitis. He had concomitant ischemic heart disease and was taking prednisolone 10 mg daily and aspirin 80 mg daily. His dialysis prescription was three 2 L exchanges of 2.5% dextrose bags per day with a calcium concentration of 3.5 mEq/L (Ultrabag, Baxter International Inc.). He achieved an average weekly KT/V of over 1.7, and peritoneal equilibration testing showed that he was a “high” transporter. He produced 500 ml of urine a day. He presented to us in August 2015 with fever, abdominal pain, and turbid dialysis effluent. Physical examination revealed a blood pressure of 140/80 mmHg and a pulse rate of 90 beats/minute. His body surface area was 1.61 square meters. The abdomen was diffusely tender. The exit site was clean and healthy-looking.

Blood work on admission was unremarkable for liver function, electrolytes, and blood counts. Microscopic examination of the effluent revealed more than 1000 leucocytes/mm^3^ (our laboratory did not give absolute values for counts over 1000), predominantly neutrophils (90%). Gram staining of the effluent did not reveal organisms. Blood culture was negative.

Empirical cefazolin (1 g/2 L dialysate loading and then 250 mg/2 L dialysate three times per day) and gentamicin (40 mg/2 L dialysate daily) were given via the intraperitoneal (IP) route. Owing to a lack of clinical response on as late as the third day of admission, empirical IP vancomycin (2 g/2 L dialysate, or 35 mg/kg body weight/2 L dialysate, once weekly), IP ceftazidime (1 g/2 L dialysate loading and then 250 mg/2 L dialysate three times per day), and IP amikacin (120 mg/2 L dialysate, or 2 mg/kg/2 L dialysate, once daily) were given to achieve broader antimicrobial coverage. In addition, fungal prophylaxis was given in the form of oral nystatin syrup at a dose of 500,000 units four times a day. Vancomycin levels were not checked. Clinical remission was achieved two days later as evidenced by clearing of the effluent and the disappearance of leucocytes on microscopy. Later, effluent bacterial culture came back with *Listeria monocytogenes*, for which the minimum inhibitory concentration of penicillin was 0.5 *μ*g/ml. Our microbiology laboratory reported susceptibility to only penicillin for *Listeria* strains.

Accordingly, the antibiotic regime was changed to IP ampicillin (250 mg/2 L dialysate three times per day) and amikacin for 4 weeks despite clinical resolution at the time of starting IP ampicillin.

## 3. Discussion

Peritoneal dialysis-related peritonitis caused by *Listeria monocytogenes* is rare. To date, only 19 cases have been reported (Table 1) [3–20]. A high index of suspicion is required for timely diagnosis, especially in patients who are immunocompromised or have other risk factors [2]. As regards to treatment for *Listeria* infections in general, ampicillin is considered first-line therapy [21]. Trimethoprim/sulphamethoxazole can be considered if contraindications to ampicillin exist, such as allergies [3, 22]. Adding an aminoglycoside can enhance bacterial clearance because ampicillin alone is bacteriostatic [23]. Cephalosporins are generally ineffective [24]. While vancomycin is effective against Gram-positive bacteria, its limited ability to penetrate the eukaryote cell membrane makes it ineffectual against intracellular bacteria, such as *Listeria* species. Indeed, vancomycin failure in the context of *Listeria monocytogenes* PD-related peritonitis has been widely reported: In 6 of the reported cases where vancomycin was part of the primary regime, the only case that ended in resolution used amoxicillin as well. Vancomycin failed to clear the infection in the remaining 5 cases, even when used in combination with an aminoglycoside, a cephalosporin, or trimethoprim-sulphamethozazole (Table 1). On the other hand, the reported cases are strongly in favour of ampicillin/amoxicillin as either primary or salvage therapy: intraperitoneal ampicillin/amoxicillin with or without an aminoglycoside resulted in cure in 7 of the reported cases. Oral or intravenous ampicillin or amoxicillin resulted in cure in 5 cases. It is for the abovementioned reasons that we substituted ceftazidime and vancomycin with ampicillin while keeping the amikacin despite clinical resolution of the peritonitis following therapy with vancomycin, ceftazidime, and amikacin.

There is some concern regarding the use of ampicillin through the intraperitoneal route, as some authors have shown that ampicillin has little in vitro antibacterial activity against Enterococci when added to common peritoneal dialysates [25]. In another study, Szeto et al. showed that oral amoxicillin could effectively treat Enterococcal PD-related peritonitis [26]. Whether this holds true for *Listeria monocytogenes* peritonitis remains to be proven.

In conclusion, *Listeria monocytogenes* PD-related peritonitis should be treated with an ampicillin- or amoxicillin-based regime with or without the addition of an aminoglycoside. While the majority of reported cases used the intraperitoneal route of administration, it is as yet uncertain whether oral or intravenous administration of ampicillin or amoxicillin might be an equal or better alternative.

## Figures and Tables

**Table 1:** Nineteen reported cases of CAPD-related peritonitis caused by *Listeria monocytogenes.*

Year	Presenting symptoms	Immunocompromise/details (if any)	Antibiotic therapy	Duration of treatment	Outcome	References
1983	Abdominal pain	Immune thrombocytopenic purpura	IV and IP erythromycin and IV trimethoprim/sulphamethoxazole	Unknown	Resolution	[3]
1989	Abdominal pain	SLE on prednisolone and azathioprine	Ampicillin and gentamicin (route unknown)	4 weeks	Resolution	[4]
1989	Abdominal pain	SLE	Vancomycin (route unknown) and then IV ampicillin	N/A	Resolution (vancomycin failure)	[5]
1990	Abdominal pain	Wegener's granulomatosis on cyclophosphamide 25 mg daily	IP ampicillin and oral pivampicillin	3 weeks	Resolution	[6]
1991	Abdominal pain and fever	Chronic lymphocytic leukaemia on prednisolone	Oral amoxicillin and IV gentamicin	4 weeks	Resolution (vancomycin failure)	[7]
1991	Abdominal pain	Cirrhosis	Ampicillin (route unknown)	Unknown	Resolution (vancomycin failure)	[8]
1992	Abdominal pain, diarrhea, and nausea	History of kidney transplantation	IV tobramycin and IP ampicillin	2 weeks	Resolution	[9]
1994	Abdominal pain	Polymyositis	IP ampicillin and gentamicin	N/A	Resolution (vancomycin failure)	[10]
2002	Abdominal pain and nausea	SLE	IP cephalosporins and ampicillin	3 weeks	Resolution	[11]
2003	Septic shock	SLE on prednisolone 5 mg daily and azathioprine 50 mg daily	IV ampicillin and amikacin	4 weeks	Resolution	[12]
2008	Abdominal pain, cloudy effluent, and fever	Congestive heart failure	Vancomycin and netilmicin	N/A	Resolution of peritonitis but death from heart failure (after prolonged therapy)	[13]
2011	N/A	Hypertension	IV amoxicillin	N/A	Resolution	[14]
2011	N/A	Chronic glomerulonephritis	IP amoxicillin	N/A	Resolution	[14]
2011	N/A	Congestive heart failure	Vancomycin and cetazidime and IP gentamycin	N/A	Resolution (vancomycin failure)	[15]
2016	Abdominal pain, fever, nausea, and vomiting	Intake of Faroese salmon	Oral amoxicillin and IP vancomycin	Amoxicillin for 2 weeks and vancomycin for 3 weeks	Resolution	[16]
2016	Septic shock	Congestive heart failure	IP ampicillin and gentamicin	N/A	Resolution	[17]
2017	Abdominal pain, diarrhea, anorexia, and fatigue	HIV	IP ampicillin	2 weeks	Resolution	[18]
2017	Fever, cloudy effluent, and meningitis	Diabetes mellitus	Failure to respond to IP vancomycin and ceftazidime and trimethoprim-sulphamethoxazole	N/A	Vancomycin failure and death	[19]
2017	Septic shock	Chronic glomerulonephritis, alcoholic cirrhosis; intake of goat curd	IP vancomycin and amikacin	N/A	Vancomycin failure and death	[20]

CAPD, continuous ambulatory peritoneal dialysis; IV, intravenous; IP, intraperitoneal; SLE, systemic lupus erythematosus; HIV, Human Immunodeficiency Virus.

## Data Availability

Underlying data supporting the results of our study can be obtained by emailing the corresponding author of this article.

## References

[1] Ramaswamy V., Cresence V. M., Rejitha J. S. (2007). Listeria—review of epidemiology and pathogenesis. *Journal of Microbiology, Immunology and Infection*.

[2] Charlier C., Perrodeau É., Leclercq A. (2017). Clinical features and prognostic factors of listeriosis: the MONALISA national prospective cohort study. *The Lancet Infectious Diseases*.

[3] Myers J. P., Peterson G., Rashid A. (1983). Peritonitis due to Listeria monocytogenes complicating continuous ambulatory peritoneal dialysis. *Journal of Infectious Diseases*.

[4] Korzets A., Andrews M., Campbel A., Feehally J., Walls J., Prentice M. (1989). Listeria monocytogenes peritonitis complicating CAPD. *Peritoneal Dialysis International: Journal of the International Society for Peritoneal Dialysis*.

[5] Allais J. M., Cavalieri S. J., Bierman M. H., Clark R. B. (1989). Listeria monocytogenes peritonitis in a patient on continuous ambulatory peritoneal dialysis. *The Nebraska Medical Journal*.

[6] Al-Wali W. I., Baillod R., Hamilton-Miller J. M., Kyi M. S., Brumfitt W. (1990). Listeria monocytogenes peritonitis during continuous ambulatory peritoneal dialysis (CAPD). *Postgraduate Medical Journal*.

[7] Dryden M. S., Jones N. F., Phillips I. (1991). Vancomycin therapy failure in Listeria monocytogenes peritonitis in a patient on continuous ambulatory peritoneal dialysis. *Journal of Infectious Diseases*.

[8] Hart K. A., Reiss‐Levy E. A., Trew P. A. (1991). Listeria monocytogenes peritonitis associated with CAPD. *Medical Journal of Australia*.

[9] Lunde N. M., Messana J. M., Swartz R. D. (1992). Unusual causes of peritonitis in patients undergoing continuous peritoneal dialysis with emphasis on Listeria monocytogenes. *Journal of the American Society of Nephrology: JASN*.

[10] Banerji C., Wheeler D. C., Morgan J. R. (1994). Listeria monocytogenes CAPD peritonitis: failure of vancomycin therapy. *Journal of Antimicrobial Chemotherapy*.

[11] Ahmad M., Krishnan A., Kelman E., Allen V., Bargman J. M. (2008). Listeria monocytogenes peritonitis in a patient on peritoneal dialysis: a case report and review of the literature. *International Urology and Nephrology*.

[12] Tse K. C., Li F. K., Chan T. M., Lai K. N. (2003). Listeria monocytogenes peritonitis complicated by septic shock in a patient on continuous ambulatory peritoneal dialysis. *Clinical Nephrology*.

[13] Stylianou K., Passam A., Papoutsakis A., Perakis K., Kroustalakis N., Daphnis E. (2008). Listeria monocytogenes peritonitis in a peritoneal dialysis patient with severe heart failure. *Kidney*.

[14] Bierhoff M., Krutwagen E., van Bommel E. F., Verburgh C. A (2011). Listeria peritonitis in patients on peritoneal dialysis: two cases and a review of the literature. *The Netherlands Journal of Medicine*.

[15] Benjelloum O., Sánchez Álvarez J. E., Rodríguez Suárez C. (2011). Listeria monocytogenes: an infrequent cause of peritonitis in peritoneal dialysis. *Nefrologia*.

[16] Poulsen H. B., Steig T. A., Björkman J. T., Gaini S. (2018). Peritonitis with Listeria monocytogenes in a patient on automated peritoneal dialysis. *BMJ Case Reports*.

[17] Moscovici A., Kogan M., Kliers I., Kukuy O., Segal G. (2016). Listeria peritonitis in a patient treated with peritoneal dialysis. *The Israel Medical Association Journal: IMAJ*.

[18] Sia C., Wilson S., Ananda-Rajah M., Mills J., Aung A. K. (2017). Listeria monocytogenes peritonitis in an HIV-infected patient. *Clinical Nephrology*.

[19] Beckerleg W., Keskar V., Karpinski J. (2017). Peritonitis as the first presentation of disseminated listeriosis in a patient on peritoneal dialysis-a case report. *Peritoneal Dialysis International: Journal of the International Society for Peritoneal Dialysis*.

[20] Mitrović M., Đurić P., Janković A. (2017). Unusual Listeria monocytogenes peritonitis in peritoneal dialysis patient with liver cirrhosis: a case report and review of literature. *CEN Case Reports*.

[21] Jones E. M., MacGowan A. P. (1995). Antimicrobial chemotherapy of human infection due to Listeria monocytogenes. *European Journal of Clinical Microbiology & Infectious Diseases*.

[22] Spitzer P. G., Hammer S. M., Karchmer A. W. (1986). Treatment of Listeria monocytogenes infection with trimethoprim-sulfamethoxazole: case report and review of the literature. *Clinical Infectious Diseases*.

[23] Azimi P. H., Koranyi K., Lindsey K. D. (1979). Listeria monocytogenes: synergistic effects of ampicillin and gentamicin. *American Journal of Clinical Pathology*.

[24] Krawczyk-Balska A., Markiewicz Z. (2016). The intrinsic cephalosporin resistome of Listeria monocytogenes in the context of stress response, gene regulation, pathogenesis and therapeutics. *Journal of Applied Microbiology*.

[25] Kussmann M., Schuster L., Zeitlinger M. (2015). The influence of different peritoneal dialysis fluids on the in vitro activity of ampicillin, daptomycin, and linezolid against Enterococcus faecalis. *European Journal of Clinical Microbiology & Infectious Diseases*.

[26] Szeto C. C., Ng J. K.-C., Chow K. M. (2017). Treatment of enterococcal peritonitis in peritoneal dialysis patients by oral amoxicillin or intra-peritoneal vancomcyin: a retrospective study. *Kidney and Blood Pressure Research*.

